# Cystoid macular edema associated with iridocorneal endothelial syndrome: a case report

**DOI:** 10.1186/s12886-016-0333-y

**Published:** 2016-09-01

**Authors:** Keita Suzuki, Tadashi Mizuguchi, Yui Seno, Atsuhiro Tanikawa, Masayuki Horiguchi

**Affiliations:** Department of Ophthalmology, Fujita Health University School of Medicine, 1-98 Dengakugakubo, Kutsukake-cho, Toyoake City, Aichi 470-1192 Japan

**Keywords:** Iridocorneal endothelial syndrome, Macular edema, Dyscoria, Abnormal corneal endothelium, Non-steroidal anti-inflammatory drug, Topical nepafenac, Case report

## Abstract

**Background:**

Iridocorneal endothelial (ICE) syndrome occurs mainly in young and middle-aged women and typically presents as a unilateral disease characterized by abnormalities of the iris and corneal endothelium. While the ICE syndrome is known to be associated with glaucoma and bullous keratopathy, to our knowledge, only two cases of ICE syndrome complicated with cystoid macular edema (CME) have been reported to date. In this paper, we report a case of ICE syndrome complicated with CME treated at our institution.

**Case presentation:**

The subject was a 51-year-old woman. In October 2013, she was examined by a primary care physician for blurred vision in her left eye. Dyscoria and abnormality of the corneal endothelium were observed, and the patient was diagnosed with ICE syndrome. In November of the same year, she was referred to our institution with a decrease in visual acuity and CME, both in her left eye. At initial examination, her best corrected decimal visual acuity was 1.0 (Snellen equivalent: 20/20) in the right eye and 0.5 (20/40) in the left eye. Intraocular pressure was 12 mmHg in both eyes. She was diagnosed with Cogan–Reese syndrome based on marked ectropion uveae, peripheral anterior synechia, and abnormalities of the corneal endothelium. Marked CME was observed on ophthalmoscopy and optical coherence tomography. A topical non-steroidal anti-inflammatory drug (nepafenac 0.1 %) was applied to the left eye four times daily from January 2014. Four weeks later, the CME had resolved and her visual acuity was 1.0 (20/20).

**Conclusion:**

While non-steroidal anti-inflammatory drugs and steroids did not appear to be effective in two previously reported cases of ICE syndrome complicated with CME, topical nepafenac was effective in this case. However, more such cases are needed before concluding that topical nepafenac is effective in this situation.

## Background

Iridocorneal endothelial (ICE) syndrome, first reported by Eagle and Yanoff in 1979 [1], is a disease characterized by abnormalities of the iris and the corneal endothelium, and mainly occurs unilaterally in young and middle-aged women. It is divided into the following three subtypes based on examination findings for the iris and cornea: progressive essential iris atrophy (corectopia, iris atrophy or iris hole), Chandler syndrome (corneal edema with mild to absent iris change), and Cogan–Reese syndrome (nodular pigmented lesion of the iris). On a pathological level, epithelialized abnormal corneal endothelial cells migrate from the trabecular meshwork into the iris plane [2], leading to angle-closure glaucoma [3] and bullous keratopathy [4]. Although various theories exist on the probable cause, such as viral infection, abnormal proliferation of neural crest cells, and ectopic embryonic ocular surface epithelium, the actual cause is yet to be ascertained. Only two reports have documented ICE syndrome complicated with cystoid macular edema (CME) [5, 6], and an effective treatment has not yet been reported. In this case, we treated the patient with ICE syndrome complicated by CME with topical nepafenac, a non-steroidal anti-inflammatory drug (NSAID), and the findings are detailed here.

## Case presentation

This case report adheres to the CARE guidelines and methodology [7]. The subject was a 51-year-old woman who presented with the chief complaint of blurred vision in her left eye. Her medical and family history was unremarkable. In October 2013, she consulted a primary care physician for the blurred vision in her left eye. Her best corrected decimal visual acuity (BCVA) was 1.0 (Snellen equivalent: 20/20) in the right eye and 0.5 (20/40) in the left eye. Intraocular pressure was 13 mmHg in both eyes. Examination revealed dyscoria of the left eye, and specular microscopy showed an abnormality of the corneal endothelium (dark area within endothelial cells), leading to a diagnosis of ICE syndrome. In November of the same year, the patient experienced a further decrease in the visual acuity of her left eye and visited the physician again. Her BCVA in the left eye was 0.4 (20/50). The physician found CME using optical coherent tomography (OCT); she was referred to our institution for detailed examination and treatment.

**Fig. 1:**
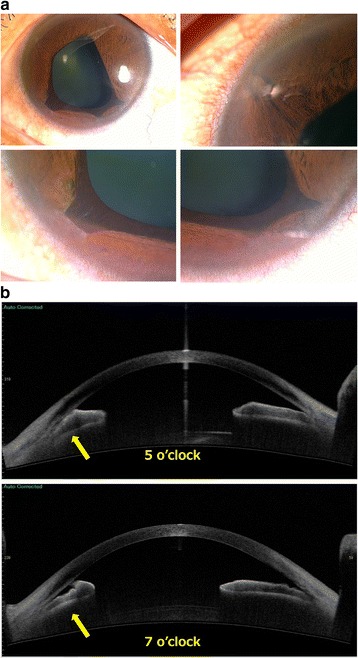
**a** Photograph of the anterior segments of the left eye. Corectopia with ectropion uveae and bridging at 4, 5, and 7 o’clock and anterior synechia at 11 o’clock. **b** Optical coherence tomography image of the anterior segments of the left eye. Anterior synechia observed at 5 and 7 o’clock

**Fig. 2:**
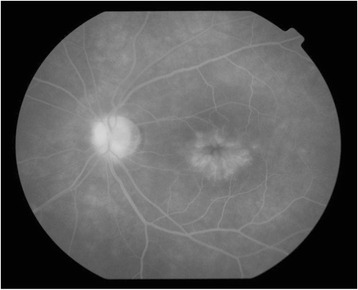
Fluorescein angiogram of the left eye. Hyperfluorescence is observed in the macular region

**Fig. 3:**
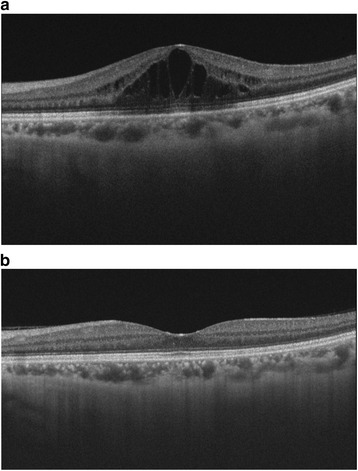
**a** Optical coherence tomography image of the macula of the left eye at the initial visit. Cystoid macular edema is observed. **b** Optical coherence tomography image of the macula of the left eye after 4 months of topical nepafenac. Cystoid macular edema has resolved

Initial examination findings were as follows. BCVA was 1.0 (20/20) in the right eye and 0.5 (20/40) in the left eye. Intraocular pressure was 12 mmHg in the right eye and 13 mmHg in the left eye. No abnormalities were observed in the anterior segment or ocular media of the right eye. In the left eye, corectopia with ectropion uveae and bridging at 4, 5, and 7 o’clock and anterior synechia at 11 o’clock were observed (Fig. 1a). OCT of the anterior segment revealed ectropion uveae and bridging at 5 and 7 o’clock with adhesion between the iris and the endothelial side of the cornea (Fig. 1b). Specular microscopy revealed an irregular rippled appearance of the endothelium, reduced corneal endothelial cell density, and a decreased percentage of hexagonal cells. These findings were particularly pronounced in the inferior portion where ectropion uveae and bridging were observed. Fundus photography revealed CME in the left eye. Late-phase petaloid pooling of fluorescence in the macular region of the left eye was observed with fluorescein angiography (Fig. 2). Macular edema was observed on OCT (Fig. 3a). Her blood tests did not reveal any abnormal results, and findings were negative for infections or autoimmune diseases that could cause uveitis or CME.

The patient was diagnosed with ICE syndrome based on the observation of marked ectropion uveae, peripheral anterior synechia, and abnormalities of the corneal endothelium. The subtype was classified as Cogan–Reese syndrome owing to the absence of corneal edema and iris hole formation as well as ectropion uveae and corectopia.

The patient was treated with topical nepafenac 0.1 % four times a day beginning in January 2014. The patient was reexamined 4 weeks later, at which time the CME had resolved and decimal BCVA was 1.0 (20/20). The patient was followed up every month for the next 8 months and BCVA was 1.0 (20/20) at each of these visits. Therefore, nepafenac was discontinued in September 2014, after 8 months of use, and CME did not recur.

## Conclusions

Although complications occur in patients who have ICE syndrome with angle-closure glaucoma or bullous keratopathy [3, 4], this report documents a case of complication with CME.

The common causes of CME include retinal vascular diseases, such as diabetic retinopathy, uveitis, post-intraocular surgery for conditions such as cataract, macular diseases such as vitreo-macular traction syndrome, retinitis pigmentosa, and use of eye drop formulations such as latanoprost [8, 9]. However, none of these CME-causing conditions was observed in this case, leading us to conclude that CME had arisen as a complication of ICE syndrome. Kocaoğlan et al. have discussed the proliferation of abnormal endothelial cells in the iridocorneal angle and iris plane and the subsequent contraction of the membrane of proliferated cells contributing to the collapse of the inner blood-retinal barrier as a possible mechanism for the complication of ICE syndrome with CME [5]. In addition, Fourmaux & Velasque have speculated that the mechanism of action may be similar to that in cases with CME after cataract surgery [6]. In this case, we considered the following factors as a mechanism for the onset of CME as a complication of ICE syndrome. First, increasing use of swept-source optical coherence tomography in recent years has led to indications of a connection between the vitreous pocket and Cloquet’s canal [10]. Further, in CME caused by use of an eye drop formulation of latanoprost, a prostaglandin analog used for glaucoma [9] and CME following cataract surgery [11], inflammatory material from the anterior chamber may reach the macula and lead to collapse of the inner blood-retinal barrier. Use of an eye drop formulation of diclofenac sodium (an NSAID) to prevent the development of CME after cataract surgery has proven to be effective [11]. In this case, since topical nepafenac was effective against macular edema, we believe that prostaglandin-like material derived from abnormal endothelial cells may have reached the macula and contributed to the collapse of the inner blood-retinal barrier. A topical or systemic steroid is another candidate for treatment of CME, but we used topical nepafenac, because ICE syndrome often causes glaucoma. Only one of the previous case reports documented the method of treatment [5], where both steroid and NSAID eye drops appeared to be ineffective. It is difficult to account for this discrepancy. It is possible that CME could have resolved in our patient as part of the natural history of this disorder, but previous cases did not include disappearance of CME, and we had observed our patient for 3 months without treatment and did not find improvement. Therefore, it is more likely that topical nepafenac eliminated the macular edema. This case suggests the possibility that NSAID eye drops could be effective for the treatment of CME accompanying ICE syndrome. However, we need more cases to confirm this possibility.

Finally, we need to address the issue of possible toxicity of NSAIDs to the cornea. Nepafenac-induced corneal graft melt was reported in one case with graft-versus-host disease [12], and nepafenac-associated bilateral corneal melt was reported in one case where nepafenac was used in error every 2 h [13]. Our patient and a previously reported patient treated with nepafenac had no corneal complications, but in patients with ICE who have malfunctioning endothelial cells, we have to be very careful about use of NSAIDs.

## Abbreviations

BCVA: Best corrected visual acuity
CME: Cystoid macular edema
ICE: Iridocorneal endothelial
NSAID: Non-steroidal anti-inflammatory drug
OCT: Optical coherence tomography

## References

[1] Eagle RC, Font RL, Yanoff M, Fine BS (1979). Proliferative endotheliopathy with iris abnormalities. The iridocorneal endothelial syndrome. Arch Ophthalmol.

[2] Campbell DG, Sheilds MB, Smith TR (1978). The corneal endothelium and the spectrum of essential iris atrophy. Am J Ophthalmol.

[3] Aihara M (2008). Glaucoma and iridocorneal endothelial (ICE) syndrome. Ophthalmology.

[4] Alvim PT, Cohen EJ, Rapuano CJ, Chung CW, Pereira ML, Eagle RC (2001). Penetrating keratoplasty in iridocorneal endothelial syndrome. Cornea.

[5] Kocaoğlan H, Unlü N, Kanpolat A, Yalvaç IS, Acar MA, Duman S (2005). Macular edema and iridocorneal endothelial syndrome: a case report. Cornea.

[6] Fourmaux E, Velasque L (2005). Progressive essential iris atrophy associated with chronic cystoid macular edema. J Fr Ophthalmol.

[7] Gagnier J, Kienle G, Altman DG, Moher D, Sox H, Riley DS (2013). The CARE group the CARE guidelines: consensus-based clinical case report guideline development. J Clin Epidemiol.

[8] Tsujikawa A. Cystoid macular edema. In: Ophthalmology. 2nd ed. I-J-3-8. Tokyo, Japan: Bunkodo Co. Ltd. pp. 477–8.

[9] Heier JS, Steinert RF, Frederick AR (1998). Cystoid macular edema associated with latanoprost use. Arch Ophthalmol.

[10] Kishi S (2014). Visualization of the vitreous body using swept source OCT. J Jpn Ophthalmol Assoc.

[11] Miyake K, Masuda K, Shirato S, Oshika T, Eguchi K, Hoshi H (2000). Comparison of diclofenac and fluorometholone in preventing cystoid macular edema after small incision cataract surgery: a multi centered prospective trial. Jpn J Ophthalmol.

[12] Wolf EJ, Kleiman LZ, Schrier A (2007). Nepafenac-associated corneal melt. J Cataract Refract Surg.

[13] Feiz VF, Oberg TJ, Kurz CJ, Mamalis N, Moshirfar M (2009). Nepafenac-associated bilateral corneal melt after photorefractive keratectomy. Cornea.

